# Mitochondria Are Related to Synaptic Pathology in Alzheimer's Disease

**DOI:** 10.4061/2011/305395

**Published:** 2011-09-12

**Authors:** Stavros J. Baloyannis

**Affiliations:** Department of Neurology, School of Medicine, Aristotle University of Thessaloniki, 54006 Thessaloniki, Greece

## Abstract

Morphological alterations of mitochondria may play an important role in the pathogenesis of Alzheimer's disease, been associated with oxidative stress and A**β**-peptide-induced toxicity. We proceeded to estimation of mitochondria on electron micrographs of autopsy specimens of Alzheimer's disease. We found substantial morphological and morphometric changes of the mitochondria in the neurons of the hippocampus, the neocortex, the cerebellar cortex, the thalamus, the globus pallidus, the red nucleus, the locus coeruleus, and the climbing fibers. The alterations consisted of considerable changes of the cristae, accumulation of osmiophilic material, and modification of the shape and size. Mitochondrial alterations were prominent in neurons, which showed a depletion of dendritic spines and loss of dendritic branches. Mitochondrial alterations are not related with the accumulation of amyloid deposits, but are prominent whenever fragmentation of the Golgi apparatus exists. Morphometric analysis showed also that mitochondria are significantly reduced in neurons, which demonstrated synaptic pathology.

## 1. Introduction

Alzheimer's disease (AD) is an insidiously progressive severe presenile and senile dementia, involving a number of cellular and biochemical mechanisms, affecting millions of humans as the most common cause of cognitive decline worldwide.

From the clinical point of view AD is mostly characterized by age-dependent cognitive decline affecting memory primarily, associated frequently with behavioral and mood disorders, which increasingly appear as the disease advances [1]. 

From the neuropathological point of view, Alzheimer's disease is mostly characterized by selective neuronal loss [2, 3], marked synaptic alterations [4–6], morphological mitochondrial abnormalities [7, 8], tau pathology [9] resulting in neurofibrillary degeneration (NFT) [10], inflammatory responses and mainly by extracellular extensive deposits of polymers of A*β* peptide in the form of neuritic plaques [11, 12] in the neocortex, the hippocampus, and many subcortical structures, which are involved in cognitive function.

 The production and accumulation of A*β* peptide are the result of the posttranslational proteolysis of the APP [13], which is one of the defining features, as real pathological hallmark, for the neuropathological diagnosis of the disease. In addition Alzheimer's disease is characterized ultrastructurally by organelle pathology involving mostly the microtubules, the mitochondria, and Golgi apparatus [14]. 

 From the etiological point of view it would be hypothesized that the multiple genetic loci [15, 16], associated with familial Alzheimer's disease would plead in favor of the heterogeneity of the disease and support the idea that the phenomenological expression of Alzheimer's disease is the final consequence of various metabolic, neurochemical, and morphological alterations, based on a broad genetic background [17], since accumulation of A*β* peptide at synaptic terminals may be associated with synaptic damage and cognitive decline in patients with AD [18]. 

Although the majority of familial or inherited AD, which manifests at an early age, are often associated with mutations in A*β*PP [19], the vast majority of the sporadic ones, which manifests usually at later stages of life, are proved to be multifactorial, including induced expression of A*β*PP [20–22] by pathological stimuli, environmental factors, as well as deprivation of trophic factors.

 Moreover the increased risk of Alzheimer's disease in sporadic cases, when a maternal relative is afflicted with the disease pleads on the other hand in favor of a maternally derived predisposition, was related probably to mitochondrial DNA (mtDNA) [23]. Mitochondrial dysfunction on the other hand is associated with oxidative stress, which may play an important role in the early pathogenetic stages of Alzheimer's disease [24, 25], presumably prior to the onset of the cognitive dysfunction since a substantial body of evidence suggests that mitochondria play a crucial role in ageing-related neurodegenerative diseases [24].

## 2. Material and Methods

### 2.1. Patients

We studied the hippocampus, the acoustic cortex, the visual cortex, the thalamus, the globus pallidus, the locus coeruleus, the red nucleus, and many areas of the cerebellar cortex in ten brains of patients who suffered from Alzheimer's disease, four men and six women, aged 62–87 years, who fulfilled the clinical, neuropsychological, and laboratory diagnostic criteria of Alzheimer's disease. The mean education of the patients was 15.2 years, and all of them spoke their native language fluently. Screening procedures were applied included medical history, medical examination, cardiological investigation, and physical neurologic assessment, and psychiatric and neuropsychological examinations. 

 All the patients underwent EEG, carotid duplex Doppler, computerized tomography (CT) scanning and magnetic resonance imaging (MRI) of the brain, and single-photon emission computed tomography (SPECT).

The mental status of the patients was assessed by Minimental State Examination (MMSE) and dementia rating scale (DRS) [26] and ADAS-COX test.

The cause of death of the patients was heart arrest following to cardiac infarct one to seven months after the final neurological assessment.

The postmortem examination of each one of the cases was performed within 6 h after death.

### 2.2. Electron Microscopy

Small samples from the hippocampus (2 × 2 × 2 mm), the acoustic cortex, the visual cortex, the thalamus, the globus pallidus, the locus coeruleus, and from many areas of the cerebellar cortex were excised and immersed in Sotelo's fixing solution, composed of 1% paraformaldehyde, 2.5% glutaraldehyde in cacodylate buffer 0.1 M, adjusted at pH 7.35. Then they were postfixed by immersion in 1% osmium tetroxide for 30 min at room temperature and dehydrated in graded alcohol solutions and propylene oxide. 

Thin sections were cut in a Reichert ultratome, contrasted with uranyl acetate and lead citrate, and studied in a Zeiss 9aS electron microscope. 

We also studied the morphology of the mitochondria, the Golgi apparatus, and the synapses and proceeded to morphometric estimations at electron microscope on micrographs of a standard magnification of 56.000x.

### 2.3. Light Microscope, Golgi Staining, Golgi-Nissl Method

The remaining parts of the above-mentioned areas of the brain and the cerebellum were processed for silver impregnation techniques, according to rapid Golgi staining. Thus, after a four-week fixation in formalin they were immersed in potassium dichromate (7 g potassium dichromate in 300 mL water) for 10 days. Then they were immersed in 1% silver nitrate for 10 days. Following a rapid dehydration in graded alcohol solutions, the specimens were embedded in paraffin and cut, some of them at 100 *μ* and some at 25 *μ*, alternatively.

The sections of 25 *μ* were stained also with methylene blue, according to Golgi-Nissl method. All the sections were mounted in permount, between two cover slips and studied in a Zeiss Axiolab Photomicroscope.

We estimated the dendritic arborization, the number of the branches, and the dendritic spines morphometrically in light microscope in sections stained according to rapid Golgi method and Golgi-Nissl staining.

### 2.4. Statistical Analysis

Statistical analysis was based on the *t*-test on the basis of 5000 mitochondria from 30 specimens of Alzheimer's disease brains and 30 specimens of normal control brains.

## 3. Results

### 3.1. Silver Impregnation Technique

Application of the silver impregnation technique revealed neuronal loss and marked abbreviation of the dendritic arborization in all the layers of the acoustic and the visual cortex, the hippocampus, the thalamus, the globus pallidus, the locus coeruleus, the red nucleus, and the cerebellar cortex. Layer I, of the acoustic and visual cortex, which includes Cajal-Retzius cells, which normally develop very long horizontal axonic profiles [27, 28], was practically empty of neurons in the patients who suffered from Alzheimer's disease, in contrast to normal control brains. Loss of tertiary dendritic branches was also noticed in the acoustic and the visual cortex in all of the specimens. 

Abbreviation of the dendritic arborization was prominent mostly in the neurons of layers III and V of the acoustic and visual cortex, in the pyramidal neurons of the hippocampus as well as in the polyhedral neurons of the locus coeruleus and the Purkinje cells of the cerebellar cortex, which demonstrated also a marked decrease of the number of dendritic spines in comparison with the normal control brains. 

The axonic collaterals in layers III, IV, V, and VI of the acoustic and visual cortex were dramatically decreased in comparison with the normal controls.

Decrease of the branches of the apical dendrites of the cortical neurons as well as decrease in spine density was widespread phenomena seen in the large majority of the neurons of the acoustic and the visual cortex, in the hippocampus, the thalamus, the globus pallidus, the red nucleus, the locus coeruleus, and the cerebellar neurons.

### 3.2. Electron Microscopy

Electron microscopy revealed pathological alterations of the dendritic spines and impressive decrease in spine density in the secondary and tertiary dendritic branches in all the layers of the acoustic and visual cortex. 

Reduction in spine size was prominent in neurons of layers II, III, and V. A substantial number of dendritic spines demonstrated large multivesicular bodies, dysmorphic spine apparatus, and mitochondria, which were characterized by marked morphological alterations.

Morphological alterations of the dendritic spines were noticed also in the pyramidal neurons of the hippocampus, the large polyhedral neurons of the thalamus and the globus pallidus, the polyhedral neurons of the locus coeruleus as well as the Purkinje cells of the cerebellar hemispheres. Giant spines were seen mostly in the hippocampus and in the Purkinje cells of the cerebellum. 

**Figure 1:**
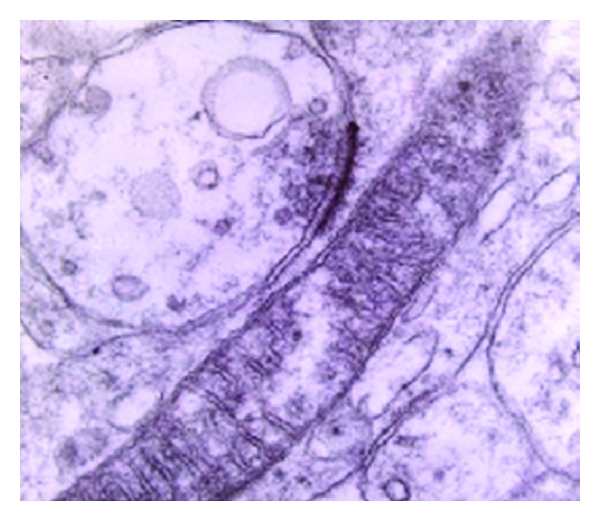
Dendritic profile of a Purkinje cell in a case of Alzheimer's disease including elongated mitochondrion, showing disruption of the cristae. The presynaptic profile, presumably a terminal of parallel fiber is characterized by the marked poverty of the synaptic vesicles (mag. 65.000x).

In large number of presynaptic terminals in the acoustic and the visual cortex of the patients who suffered from Alzheimer's disease, the ultrastructural study revealed an impressive polymorphism and pleomorphism of the synaptic vesicles, which were dramatically decreased in number in comparison with normal control brains (Figure 1).

**Figure 2:**
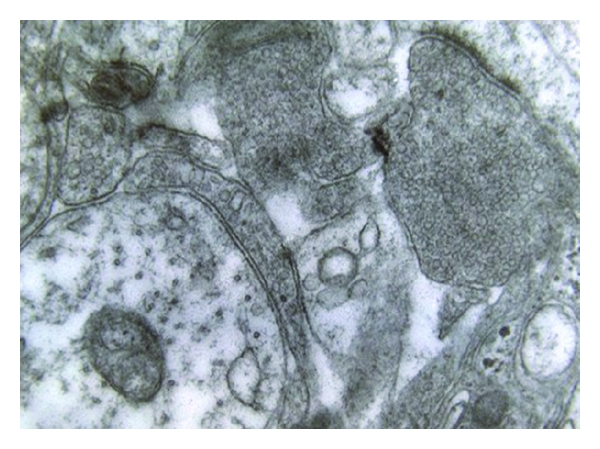
Mossy fibers of the cerebellar cortex in a case of Alzheimer's disease showing decrease of the number of the synaptic vesicles and lack of mitochondria (mag. 65.000x).

Impressive poverty of the synaptic vesicles was particularly seen in the presynaptic terminals in layers III, IV, and V of the acoustic and visual cortex as well as in the mossy fibers of the cerebellar cortex (Figure 2). Decrease of the number of synaptic vesicles and marked polymorphism of the remained vesicles was also seen in the hippocampus the thalamus, the locus coeruleus, and in the parallel and climbing fibers of the cerebellar cortex.

Mitochondrial pathology was seen in the majority of the dendritic spines in all of the specimens, which consisted of substantial change of shape and size, fragmentation of cristae, and accumulation of osmiophilic material in a considerable number of mitochondria. 

Many dendritic profiles contained mitochondria, which showed an impressive polymorphism in the arrangement of the cristae, which sometimes showed a concentric configuration or in other places they were arranged in a parallel way to the long axis of the organelle. Some dendrites of Purkinje cells and a substantial number of climbing fibres contained very large elongated mitochondria. 

**Figure 3:**
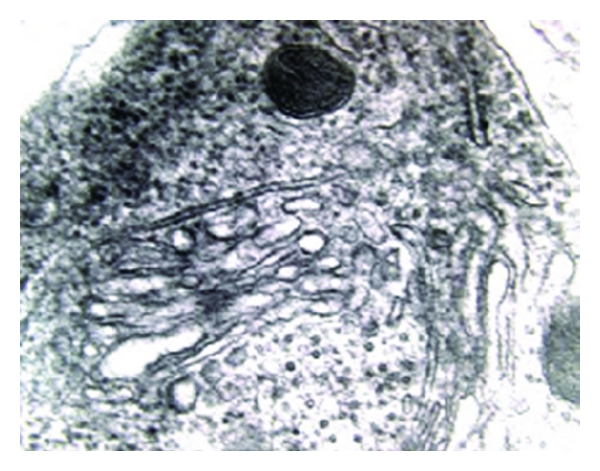
Small dense mitochondrion associated with fragmentation of the cisternae of Golgi apparatus (meg. 70.000x).

Small round mitochondria intermixed with dense bodies or associated with fragmentation of the Golgi apparatus (Figure 3) were seen in the soma of a considerable number of neurons of the visual cortex, the hippocampus, the locus coeruleus, the red nucleus, the large polyhedral neurons of globus pallidus, and the Purkinje cells of the cerebellar cortex in contrast to normal control brains, in which the mitochondria looked unremarkable.

It is worth to emphasize that morphological alterations of the mitochondria were also seen in the soma, the perivascular astrocytic processes, and the astrocytic sheaths in Alzheimer's brains in contrast to normal controls.

From the morphometric point of view the ellipsoid mitochondria in the dendritic spines of the normal control brains appear to have an average diameter of 650 ± 250 nm and a mean axial ratio of 1.9 ± 0.2. The round or global mitochondria in normal controls appeared as having a mean mitochondrial radius of 350 nm. 

In Alzheimer's disease brains, the ellipsoid mitochondria of the neurons of the acoustic and the visual cortex appear to have an average diameter of 480 ± 250 nm and a mean axial ratio of 1.7 ± 0.2. The round mitochondria have a mean radius of 280 nm.

## 4. Discussion

The mitochondria, which are the only nonnuclear constituents of the cell with their own DNA (mtDNA), having machinery for synthesizing RNA and proteins, are critical to homeostasis of the cell, by virtue of providing most of the energy for cellular processes and by their involvement in other metabolic pathways. Mitochondria are also critical regulators of cell apoptosis, as being involved in a considerable number of neurodegenerative diseases [29, 30], since it is well known that energy production, realized by oxidative phosphorylation, occurs in the mitochondria, which generate most of the cell's supply of ATP. 

From the morphological point of view the shape and size of the mitochondria as well are highly variable [31], depending on fission and fusion [32]. Their morphology is sometimes controlled by cytoskeletal elements, namely the neurofilaments and the microtubules [33]. The change of the shape of the mitochondria occurs mostly through their move to axons, dendrites, and synaptic terminals via anterograde transport [34]. 

During the various neuronal processes approximately one-third of the mitochondria are in motion along microtubules and actin filaments [35–37], whereas the majority of them are stationary. Mitochondrial motility and accumulation are coordinated, since mitochondria are transported to regions where ATP consumption and necessity for energy are particularly high, as it takes place in the synapses, which have high energy demand for serving neuronal communication [38]. 

Mitochondrial alterations and dysfunction have been reported in several neurodegenerative diseases [39–41] associated mostly with oxidative damage [42] and vascular lesions [43]. Oxidative stress is mostly associated with amyloid *β* (A*β*) accumulation in the neocortex [7, 44, 45], playing therefore an important role in the pathogenetic mechanisms of Alzheimer's disease [46], since it is not only involved in damage to the proteins of NFT [36] and the formation of senile plaques but also involves extensive damage to the cytoplasm of neuronal populations vulnerable to death during AD [47]. 

It is also well documented that A*β* peptide may increase mitochondrial reactive oxygen species (ROS) production [48], causing further impairment of mitochondrial function [49] since the lack of histones in mitochondrial DNA renders them a vulnerable target to oxidative stress.

In all major examples of these diseases there is strong evidence that mitochondrial dysfunction occurs early and acts causally in disease pathogenesis. Mutations in mitochondrial DNA and oxidative stress, on the other hand, may contribute to ageing, which is the substantial biological background for the majority of the neurodegenerative diseases [50]. Mitochondrial dysfunction has been associated with energy crisis of the cell and excitotoxic cell death and is considered to be of substantial importance in the cascade of phenomena, which eventually lead to apoptosis. 

 Some observations in early cases of Alzheimer's disease [51] indicate that morphological alterations of the mitochondria and oxidative damage may be one of the earliest events in Alzheimer's disease. The morphological alteration of the mitochondria seen in subcortical centres, such as in the thalamus, the globus pallidus, the red nucleus, and the locus caeruleus, pleads in favor of a generalized mitochondrial dysfunction in Alzheimer's disease, which may be associated with wide neuronal loss and synaptic alterations, seriously affecting consequently, the mental faculties, which are basically related to extensive neural networks [52]. Moreover, an impressive number of disease-specific proteins interact with mitochondria. Well-documented studies [53] demonstrate that a significant amount of the N-terminal domain of APP targeted the mitochondria of cortical neuronal cells and select regions of the brain of a transgenic mouse model for AD. The accumulation of transmembrane-arrested APP blocked protein translocation, disrupted mitochondrial function, and impaired brain energy metabolism. In Alzheimer's disease the amyloid precursor protein has been localized to mitochondria as has the toxic amyloid beta peptide. The binding site for amyloid beta has been identified as alcohol dehydrogenase in the matrix space of the organelle. Many morphological alterations of AD could very well be linked to mitochondria changes since blockage of mitochondrial energy production shifts amyloid protein precursor metabolism to the production of more amyloidogenic forms of amyloid [54]. In addition amyloid beta peptide promotes permeability transition pore in brain mitochondria [55, 56]. 

 It is important to mention that many protein systems are also essential in mitochondrial function, their morphological integrity and in binding to the cytoskeleton [57]. Mitochondrial porin is an outer-membrane protein that forms regulated channels (Voltage-Dependent Anionic Channels) between the mitochondrial intermembrane space and the cytosol. Porin may play an important role in binding to neurofilaments and microtubules [37], since porin-rich domains contain most of the binding sites for MAP2 [58]. In addition preselinin-2 modulates endoplasmic reticulum-mitochondrial interactions [59], a fact that pleads in favour of the crucial role that mitochondria play in the pathogenetic cascade of Alzheimer's disease. 

The number of the mitochondria varies, according to energy state of the cell. Some evidence suggests that the mitochondria redistribute towards the dendritic profiles in response to stimulation as a manifestation of synaptic plasticity [60]. Normally a limited number of dendritic spines contain mitochondria, which are mostly small and round, been increased in number inside the dendritic branches during the synaptogenesis. A decrease in energy metabolism and altered cytochrome c oxidase (CytOX) activity are among the earliest detectable defects in AD [61, 62], affecting presumably neuronal plasticity and synaptogenesis. Some observations suggest that mitochondrial cytochrome c oxidase may be inhibited by a dimeric conformer of A*β*35, a phenomenon which emphasizes the role of the A*β* peptide on the mitochondrial dysfunction in Alzheimer's disease [63]. 

Therapies targeting basic mitochondrial processes, such as energy metabolism or free-radical generation, or specific interactions of disease-related proteins with mitochondria, hold great promise. On the basis of the mitochondrial pathology, in the pathogenetic spectrum in Alzheimer's disease, new strategies inducing protection to mitochondria by the administration of efficient antioxidant factors could be introduced in the treatment of early cases of Alzheimer's disease.
